# Redox status in the sentinel lymph node of women with breast cancer

**DOI:** 10.1080/03009734.2017.1403522

**Published:** 2017-12-21

**Authors:** María Jesús Ramírez-Expósito, Nieves Urbano-Polo, Basilio Dueñas, Joaquín Navarro-Cecilia, César Ramírez-Tortosa, María Dolores Martín-Salvago, José Manuel Martínez-Martos

**Affiliations:** a Experimental and Clinical Physiopathology Research Group CTS-1039, Department of Health Sciences, School of Experimental and Health Sciences, University of Jaén, Jaén, Spain;; b Unit of Breast Pathology, Complejo Hospitalario de Jaén, Jaén, Spain;; c Department of Pathology, Complejo Hospitalario de Jaén, Jaén, Spain

**Keywords:** Breast cancer, catalase, glutathione peroxidase, GSH, OSNA, sentinel lymph node, superoxide dismutase, total antioxidant capacity

## Abstract

**Background:**

Lymphatic metastasis is regulated in multiple steps including the transit of tumor cells via the lymphatic vessels and the successful seeding in draining lymph nodes. Thus, several molecular signals and cellular changes must be involved in this complex process to facilitate tumor cell entry, colonization, and survival in the lymph node. To our knowledge, the present work explores, for the first time in the literature, the redox status (oxidative stress parameters and enzymatic and non-enzymatic antioxidant defense systems) in the sentinel lymph node (SLN) of women with breast cancer.

**Patients and methods:**

SLNs from 75 women with breast cancer were identified using the one-step nucleic acid amplification (OSNA) method as negative (*n* = 43), with micrometastases (*n* = 13), or with macrometastases (*n* = 19). It will allow us to gain knowledge about the pro-oxidant/antioxidant mechanisms involved in the processes of distant metastases in breast cancer and also to assess whether these parameters may be alternative techniques for staging.

**Results:**

We found different levels of lipid peroxidation in SLNs with micrometastases (increased) and macrometastases (decreased), a decrease in carbonyl group content in SLNs with macrometastases only, and an increase in total antioxidant capacity (TAC) in SNLs with micrometastases and macrometastases. A decrease in the levels of reduced glutathione (GSH) also appears in the SLNs with macrometastases only. Finally, we show increased levels of superoxide dismutase (SOD) and catalase (CAT) activity in SLNs with micrometastases and macrometastases, and decreased levels of glutathione peroxidase (GPx) activity in SNLs with macrometastases but not with micrometastases.

**Conclusions:**

Redox status of lymph node microenvironment participates in the progression of metastatic breast cancer.

## Introduction

Cancer metastasis is a multistep process that involves the dissemination of cancer cells from the primary tumor to distant organs (1,2). In this process, little is known about lymphatic metastasis. In contrast to a previous hypothesis which indicated that lymphatic metastasis is an entirely passive process, several recent reports have indicated that it is regulated in multiple steps including the transit of tumor cells via the lymphatic vessels and successful seeding in draining lymph nodes (2,3).

Metastatic status in the axillary lymph nodes is the most important prognostic factor for patients with breast cancer. However, axillary lymph node dissection is associated with significant morbidity. In contrast, sentinel lymph node (SLN) biopsy is a minimally invasive procedure that also allows accurate axillary nodal staging with less morbidity (4). In fact, SLN biopsy has been validated in early breast cancer to reflect the status of the remaining lymph nodes in the draining nodal basin, and patients with negative SLNs avoid axillary lymph node dissection (5). Intraoperative one-step nucleic acid amplification (OSNA) analysis for sentinel node biopsy in breast cancer is beginning to be used to increase the sensitivity of surgical staging through the discovery of microscopic or even cellular metastases missed on routine pathologic review (6). Therefore, increasing recognition of the importance of lymph node metastasis in cancer biology has prompted studies to unravel the cellular and molecular events involved in this complex process.

Several studies support findings that reactive species are involved in the etiology and progression of breast cancer because certain markers of oxidative stress, including lipid peroxidation products such as malondialdehyde (MDA) (7) and 8-isoprostanes (8), protein oxidation products such as carbonyls and diene conjugates (9,10), and DNA adducts (11), are frequently identified in breast cancer patients (12). In contrast, endogenous antioxidants constitute the defense mechanisms that scavenge reactive species in cells, which include non-enzyme and enzyme antioxidant defense systems such as reduced glutathione (GSH), alpha-lipoic acid, coenzyme Q, ferritin, uric acid, bilirubin, metallothionein, l-carnitine, melatonin and enzymatic superoxide dismutase (SOD), catalase (CAT), and glutathione peroxidase (GPx), respectively. Of them, SOD, CAT, and GPx are the enzymes that normally act to prevent or decrease the tissue damage caused by free radicals. SOD metabolizes free radicals and dismutates superoxide anions (O_2_^•-^) to H_2_O_2_ and protects cells against O_2_^•-^-mediated lipid peroxidation, CAT converts H_2_O_2_ into H_2_O and O_2_, and GPx reduces H_2_O_2_ and other organic peroxides. Several studies have also shown significant changes in both enzymatic and non-enzymatic antioxidant systems in women with breast cancer (12,13). However, little is known about the role of oxidative stress in lymph node metastasis. Thus, it has been described in colorectal cancer cells that the increase of intracellular reactive species is first associated with cell growth and invasion. However, a further increase inhibits cancer cell proliferation, whereas any decrease in reactive species needs to stimulate lymph node metastasis (14). On the other hand, it has been also described that breast cancer cell metastasis is due to elevated levels of reactive species (15). Therefore, the knowledge of the redox status of the SLN in women with breast cancer represents a new way to study the role of redox processes and pro-oxidant/antioxidant mechanisms involved in the mechanisms of distant metastases. Also, due to the great success of selective SLN biopsy as a diagnostic staging method, oxidative stress parameters and/or the enzymatic and non-enzymatic antioxidant defense systems involved could be useful as alternative/complementary techniques of staging in women with breast cancer. To that end, in the present report we evaluate a wide range of well known and widely validated oxidative stress parameters (lipid peroxidation, protein oxidation, and total antioxidant capacity), as well as non-enzyme (GSH) and enzyme (SOD, CAT, and GPx) antioxidant defense systems in the SLN of women with breast cancer determined as negative, with micrometastases, or with macrometastases by the OSNA method. An alteration of the normal redox balance to an antioxidant state will be described in the metastatic SLN of women with breast cancer.

## Subjects and methods

### Subjects and study design

A total of 75 women with breast cancer were recruited at the Unit of Breast Pathology at the University Hospital of Jaén. Enrollment criteria included patients with T2–3 N0 breast cancer who were not previously treated with neoadjuvant chemotherapy. Patients were evaluated before surgery and were included in the study if the axilla was negative clinically and by echography, using a 7–12 MHz lineal probe. When a suspicious node appeared, core biopsy was performed. Patients signed an informed consent for the SLN procedure. This study was approved by the Ethical Committee of the University Hospital of Jaén, and all patients signed a term of free, informed consent. Patient characterization included age at diagnosis, tumor size, tumor histology, pathologic T classification, Scarff–Bloom–Richardson grade, hormonal and HER-2/neu (human epidermal growth factor receptor 2) status, and molecular subtype.

### Identification of the sentinel lymph node, evaluation of the axilla status, and surgery

**Figure 1.**
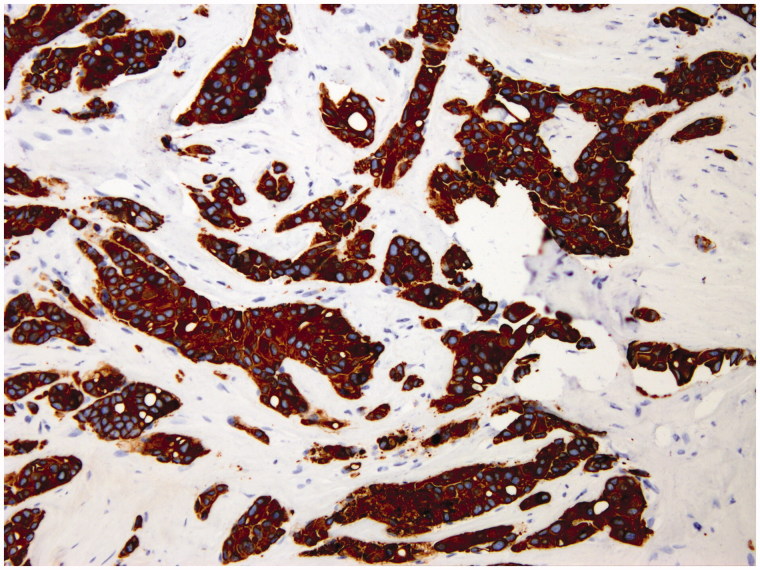
Monoclonal antibody staining for cytokeratin 19 in large-core needle biopsy of breast carcinoma obtained for diagnosis. Note the intense diffuse staining at membrane and intracytoplasmic level in 100% of tumor cells.

The sentinel procedure was performed for all patients using only a radioisotope. On the day before the surgery, lymphatic mapping was performed using 4.0 mCi of technetium-99 nanocolloid (Nanocoll, Amersham, UK) injected subareolarly. Preoperative lymphoscintigraphy was performed on all patients 1 h after the injection, and the drainage pattern was recorded. A hand-held gamma detection probe (Gamma Finder II, World of Medicine AG, Ludwigsstadt, Germany) was used to identify areas of increased activity in the axilla and nodal basins the day after the injection, usually approximately 20–24 h after the injection. The sentinel node was considered if it was radioactive or palpable. All nodes were detected at axillary level. Each sentinel node was excised, sent to the pathology department, and subjected to OSNA analysis (16). Previously, the expression of cytokeratin 19 (CK19) was confirmed in 100% of the tumor cells by large-core needle biopsy (Figure 1). The OSNA protocol consisted of homogenization of tissue in an mRNA-stabilizing solution (Lynorhag, pH 3.5; Sysmex, Barcelona, Spain) and subsequent isothermal (65 °C) amplification of CK19 using a Lynoamp amplification kit (Sysmex) through a reverse transcriptase loop-mediated isothermal amplification assay (RT-LAMP) in a gene amplification detector RD-100i (Sysmex) in compliance with the protocol described above. The technique uses six primers, which increases the specificity and speed of the reaction. Tissue homogenates from each lymph node were kept frozen at −80 °C as a back-up for possible future studies. All cases were classified according to the tumor–node–metastasis (TNM) classification of malignant tumor staging.

**Figure 2.**
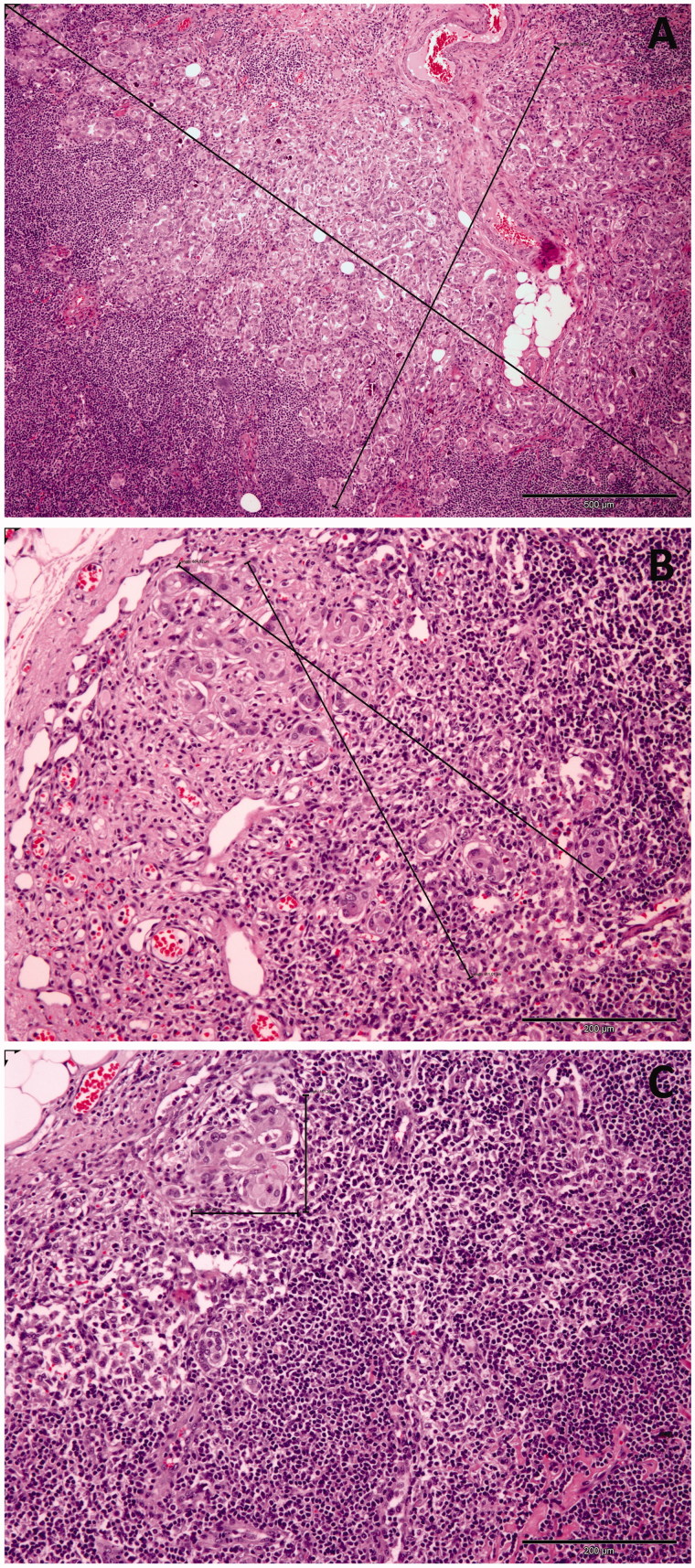
Macrometastasis, micrometastasis, and isolated tumor cells in sentinel lymph nodes of women with breast cancer. (A) H&E section (×40) shows abundant clusters of epithelial cells on a fibrous stroma at an intranodal location. The clusters are larger than 2 mm. (B) H&E section (×100) shows a focus of epithelial cells on a fibrous stroma at an intranodal location measuring more than 0.2 mm but less than 2 mm. (C) H&E section (×100) demonstrates a small cluster of epithelial cells in the subcapsular region. The cluster measures less than 0.2 mm.

In the OSNA assay, cases showing mRNA CK19 levels >250 copies/µL were considered positive and were classified as micrometastases (number of copies >250 copies/µL, <5,000 copies/µL) or macrometastases (number of copies >5,000 copies/µL) following system specifications based on previous calculations. Cases identified as ‘negative’ (<250 copies/µL) by the system were classified further as isolated tumor cells (ITCs) (number of copies/µL >100, but fewer than 250) or true negative if the number of copies/µL was <100. A complete axillary node dissection was performed in those patients with macrometastases only. Lymph nodes submitted as part of the axillary dissection were evaluated using standard H&E staining (Figure 2). Breast surgery with conservative treatment (palpable or roll lumpectomy) or mastectomy (simple, skin sparing, or nipple sparing) with immediate reconstruction was performed as planned. Results of the intraoperative assessment were recorded, and patients were followed every six months after surgery clinically and by echography.

### Oxidative stress parameter assays

#### Lipid peroxidation assay

Lipid peroxidation was measured by analyzing the amount of thiobarbituric acid reactive substances (TBARS) as previously described by Ramírez-Expósito et al. (12). Briefly, 25 µL of each sample was mixed with 100 µL of ice-cold 20% trichloroacetic acid (TCA). After centrifugation, a volume of supernatant was added to an equal volume of 0.67% 4,6-dihydroxypyrimidine-2-thiol (TBA), and the mixture was kept in a boiling water bath for 15 min. Samples were cooled to room temperature, and the absorbance at 532 nm was recorded after subtracting blanks containing TCA and TBA in an equal volume. The signal was read against a MDA standard curve, and the results were expressed as ng of MDA per mg of protein.

#### Protein oxidation assay

Protein oxidation was measured by analyzing the carbonyl group content of proteins as previously described by Ramírez-Expósito et al. (12). Briefly, 25 µL of sample was mixed with 100 µL of ice-cold 20% TCA and centrifuged. Protein precipitates were left to react with 10 mM 2,4-dinitrophenylhydrazine for an hour at room temperature in the dark. After the reaction, proteins were precipitated with 20% TCA, and unreacted dye was washed twice with 10% TCA. The pellets were dissolved in 1 M NaOH, and absorbance was recorded at 360 nm. The results were expressed as nmol per mg of protein using an extinction coefficient of 2.1 × 10^4^ M^−1^ cm^−1^.

#### Total antioxidant capacity (TAC) assay

TAC was measured using copper(II)-neocuproine as chromogenic oxidant, as previously described by Apak et al. (17) as the CUPRAC method. Results were compared with a standard curve obtained with Trolox and were expressed in nmol of Trolox equivalents per mg of protein.

### Non-enzymatic antioxidant defense system

#### Determination of reduced glutathione (GSH)

GSH levels were measured using a glutathione colorimetric assay kit from BioVision (Deltaclon, Madrid, Spain), according to the manufacturer’s instructions. Data were presented as ng of GSH per mg of protein.

### Enzymatic antioxidant defense systems

#### Superoxide dismutase (SOD) assay

SOD activity was measured according to Paoletti et al. (18): a 10 µL sample was mixed with reaction buffer containing 100 mM triethanolamide–diethanolamide buffer (TDB) pH 7.4, 7.5 mM β-nicotinamide adenine dinucleotide (NADH) and ethylenediaminetetraacetic acid (EDTA)/MnCl_2_ at a ratio of 1 : 2 (v/v). To start the reaction, 25 µL of 10 mM β-mercaptoethanol was added. The absorbance was recorded at 340 nm for 2–15 min. One unit of SOD activity was defined as the amount of enzyme necessary to produce a 50% inhibition of the NADH oxidation rate under the assay conditions.

#### Catalase (CAT) activity assay

Samples were processed and analyzed for CAT activity as described by Aebi (19) with slight modifications by Cohen et al. (20). Briefly, 10 µL of sample was added to 10 mM H_2_O_2_ in 20 mM potassium phosphate buffer (pH 7.0) and incubated at 30 °C for 1 min. Initial reaction rate was measured from the decrease in absorbance at 240 nm. CAT activity was expressed in nmol of H_2_O_2_ decomposed per minute per mg of protein under the assay conditions.

#### Glutathione peroxidase (GPx) activity assay

GPx activity was measured according to Ellerby and Bredesen (21). The reaction mixture was formed by 50 mM potassium phosphate (pH 7.4), 25 mM β-nicotinamide adenine dinucleotide phosphate (NADPH), 1 mM of GSH, and 100 U/mL of yeast glutathione reductase; 10 µL of sample was added and mixed with the reaction mixture in a 96-well dish. The hydroperoxide-independent NADPH consumption rate was recorded for 3 min at 37 °C at 340 nm. Then, 2.5 µL of tert-butyl hydroperoxide was added to start the reaction, mixed, and the overall rate at 340 nm was recorded. The same procedure was carried out in the same reaction volume without the sample. This allowed subtraction of the non-enzymatic rate of GSH oxidation. GPx activity was expressed as µmol of NADH oxidized per minute per mg of protein under the assay conditions.

### Statistical analysis

All values represent the mean ± standard error of the mean (SEM). Data were analyzed by analysis of variance (ANOVA) plus least significant difference (LSD) *post-hoc* test, using IBM SPSS v.19 software. Values of *P* < 0.05 were considered significant.

## Results

### Subject population

This study involves a population sample characterized by the clinicopathological parameters presented in Table 1. Table 2 shows the characteristics of the SLN and lymph nodes obtained after axillary dissection.

**Table 1.** Patient and tumor characteristics.

	Sentinel lymph node status					
	Negative		Micrometastases		Macrometastases	
Characteristic	*n* = 43	%	*n* = 13	%	*n* = 19	%
Age (years)						
Mean	50.3 ± 1.6		50.7 ± 3.4		51.8 ± 2.5	
Median	48.0		45.0		50.0	
Range	31–76		35–75		35–74	
Tumor histology						
Ductal	39	90.7	10	76.9	16	84.2
Lobular	3	6.9	1	7.7	3	15.8
Other	1	2.3	2	15.4	0	0.0
Molecular subtypes						
Luminal A	24	55.8	12	92.3	15	78.9
Luminal B	7	16.3	1	7.7	4	21.1
Her-2	3	6.9	0	0.0	0	0.0
Triple negative	9	20.9	0	0.0	0	0.0
Pathologic tumor size (cm)						
Mean ± SEM	3.65 ± 0.17		3.69 ± 0.36		3.42 ± 0.28	
Median	3.5		3.4		3.0	
Range	2–8		2.4–7		2.4–7.5	
Pathologic T classification						
0	0	0.0	0	0.0	0	0.0
1	0	0.0	0	0.0	0	0.0
2	39	90.7	11	84.6	17	89.5
3	4	9.3	2	15.4	2	10.5
Scarff–Bloom–Richardson grade					
I	7	16.3	3	23.1	6	31.6
II	19	44.2	7	53.8	12	63.1
III	17	39.5	3	23.1	1	5.3
Hormonal status						
ER+	31	72.1	13	100	19	100
ER−	12	27.9	0	0.0	0	0.0
PgR+	29	67.4	13	100	18	94.7
PgR−	14	32.5	0	0.0	1	5.3
HER-2/neu status						
Negative	34	79.1	12	92.3	16	84.2
Positive	9	20.9	1	7.7	3	15.8

**Table 2.** Sentinel and axillary lymph node dissection characterization.

Characteristic	Sentinel lymph node status		
	Negative	Micrometastases	Macrometastases
	*n* = 43	*n* = 13	*n* = 19
Sentinel lymph node removed			
Mean ± SEM	1.19 ± 0.60	1.38 ± 0.18	1.74 ± 0.25
Median	1	1	1
Range	1–2	1–3	1–4
Axillary nodes removed			
Mean ± SEM		15.40 ± 2.29	16.16 ± 1.60
Median		17.5	15.0
Range		5–30	6–33
Axillary nodes positive			
Mean ± SEM		2.90 ± 1.60	2.74 ± 1.02
Median		1	1
Range		0–17	0–18

### Oxidative stress parameters

**Figure 3.**
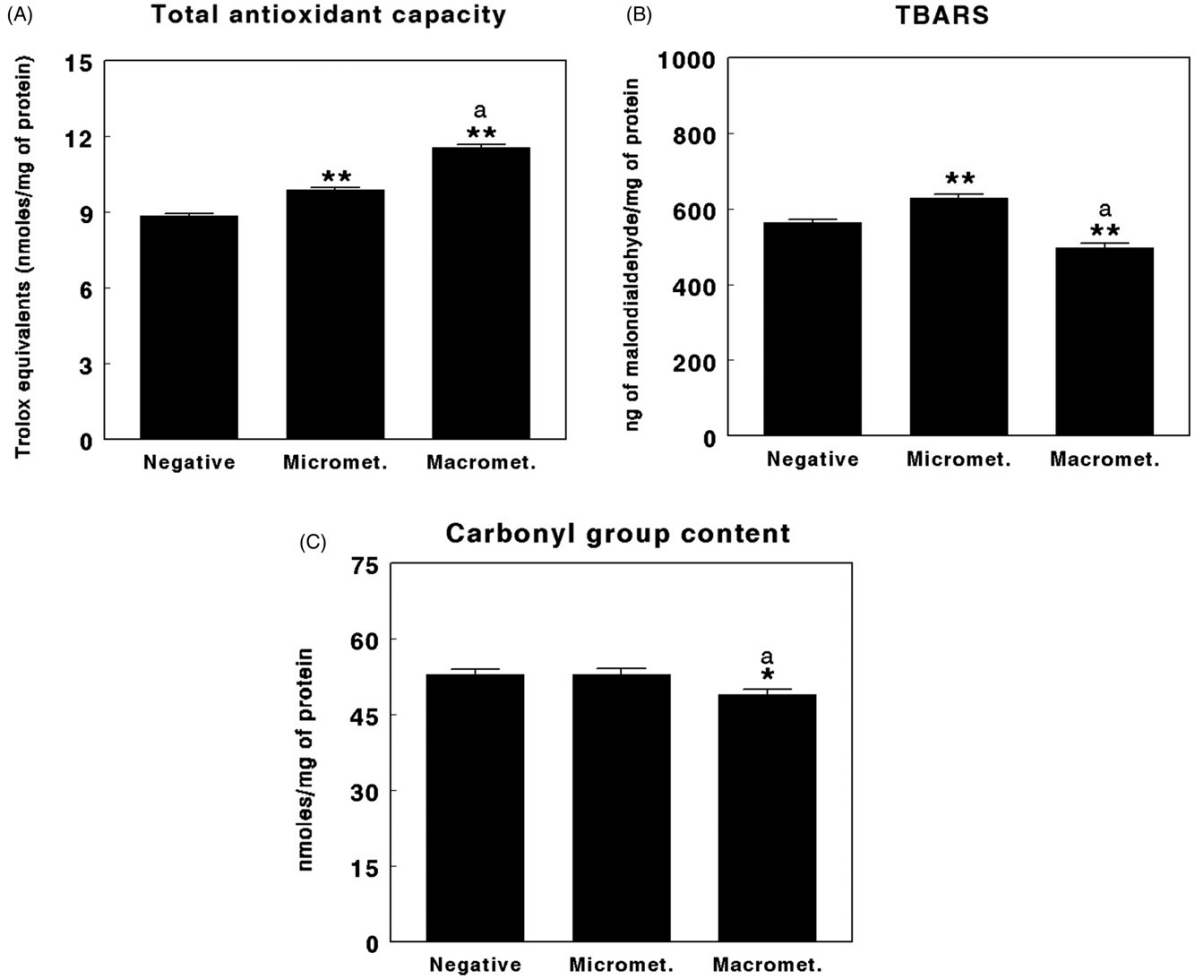
Total antioxidant capacity (TAC) (A), thiobarbituric acid reactive substances (TBARS) (B), and carbonyl group content (C) in negative sentinel lymph nodes (SLNs), SLNs with micrometastasis, and SLNs with macrometastasis of women with breast cancer. Results are expressed in nmol of Trolox equivalents per mg of protein for TAC, in ng of malondialdehyde per mg of protein for TBARS, and in nmol per mg of protein for protein carbonyls (mean ± SEM; *n* = 13–43; *P < 0.05; **P < 0.01; ^a^*P* < 0.05 micrometastasis versus macrometastasis).

**Figure 4.**
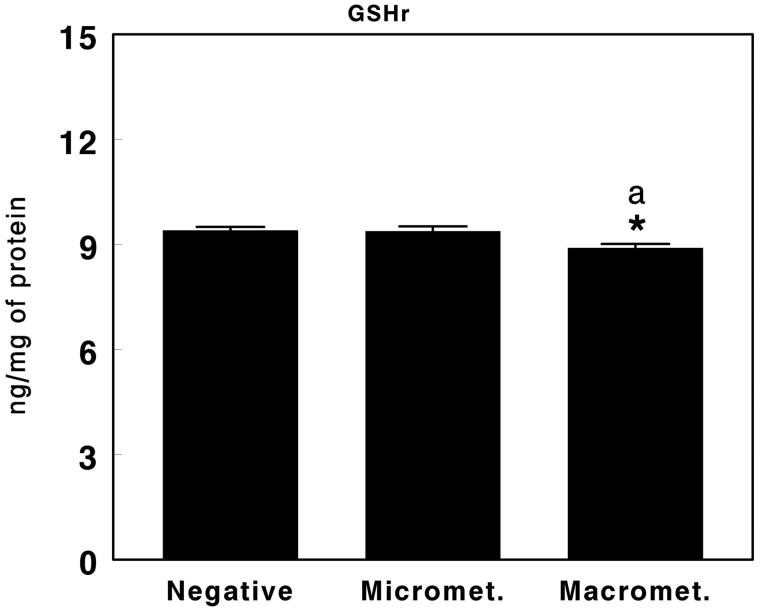
Reduced glutathione (GSH) in negative sentinel lymph nodes (SLNs), SLNs with micrometastasis, and SLNs with macrometastasis of women with breast cancer. Results are expressed in ng per mg of protein (mean ± SEM; *n* = 13–43; *P < 0.05; ^a^*P* < 0.05 micrometastasis versus macrometastasis).

**Figure 5.**
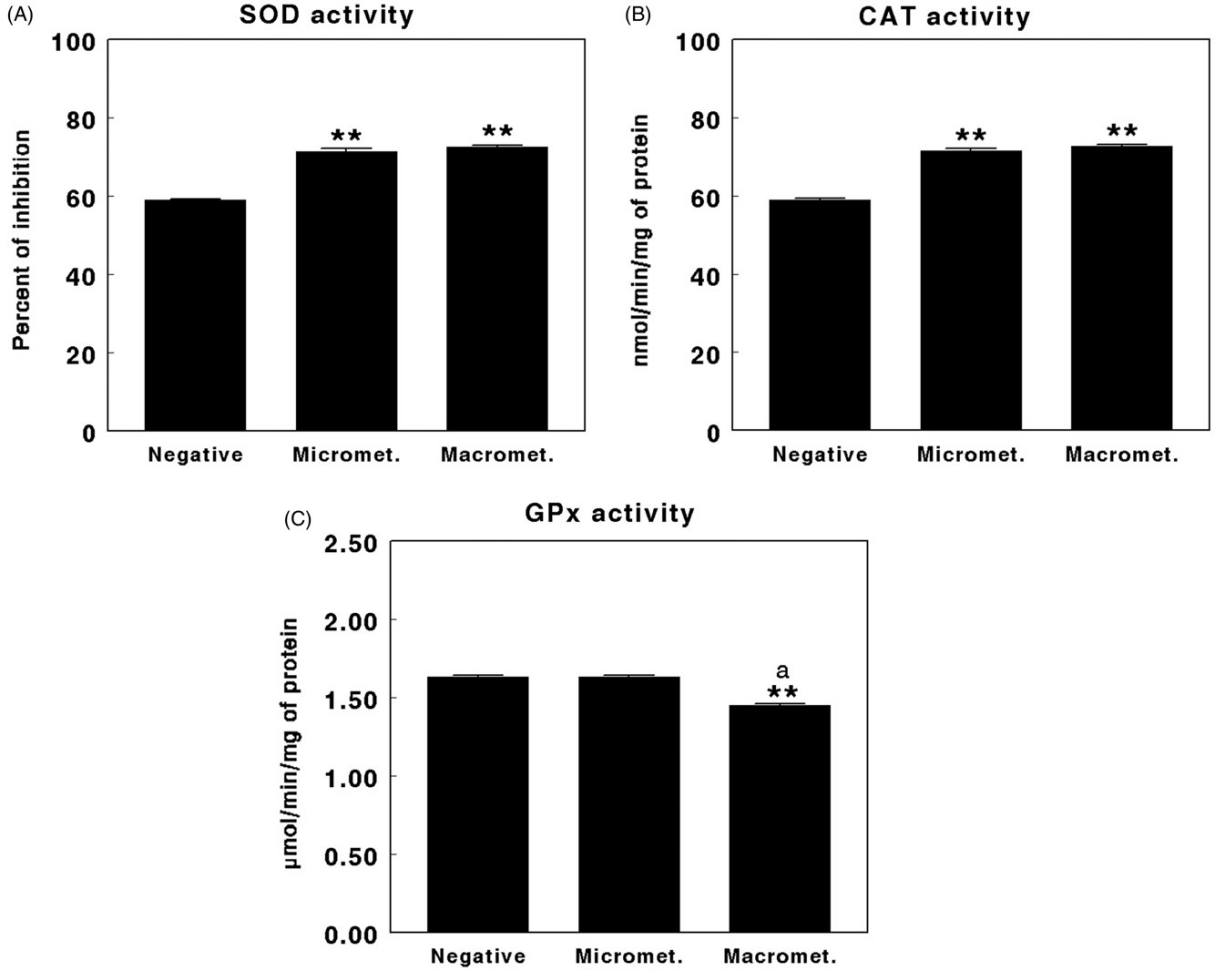
Superoxide dismutase (SOD) (A), catalase (CAT) (B), and glutathione peroxidase (GPx) (C) activity in negative sentinel lymph nodes (SLNs), SLNs with micrometastasis, and SLNs with macrometastasis of women with breast cancer. Results are expressed as percentage inhibition for SOD, in nmol per min and per mg of protein for CAT, and in µmol per min per mg of protein for GPx (mean ± SEM; *n* = 13–43; **P < 0.01; ^a^*P* > 0.05 micrometastasis versus macrometastasis).

Table 3 and Figures 3–5 show oxidative stress parameters found in the SLNs of women with breast cancer evaluated as negative, SLNs with micrometastases, or SLNs with macrometastases. Lipid peroxidation, analyzed as TBARS levels, showed significantly higher (*P* < 0.01) levels in the SLNs with micrometastases compared with negative SLNs, whereas the levels were significantly lower (*P* < 0.01) in the SLNs with macrometastases compared with negative SLNs. There were also significant differences (*P* < 0.01) between SLNs with micrometastases and macrometastases, the highest TBARS values appearing in the SLNs with micrometastases and the lowest in the SLNs with macrometastases (Figure 3(A)).

**Table 3.** Oxidative stress parameters, non-enzyme and enzyme antioxidant defense systems in the sentinel lymph node (SLN) of women with breast cancer.

	Negative SLN	SLN with micrometastases	SLN with macrometastases
Oxidative stress parameters			
Total antioxidant capacity (nmol of Trolox equivalents/mg of protein)	8.86 ± 0.09	9.88 ± 0.1	11.57 ± 0.1
Lipid peroxidation (ng of malondialdehyde/mg of protein)	564.8 ± 7.1	629.4 ± 10.2	497.5 ± 11.9
Protein oxidation (nmol per mg of protein)	53.0 ± 0.9	53.1 ± 1.06	48.9 ± 1.02
Non-enzymatic antioxidant defenses			
GSH (ng per mg of protein)	9.4 ± 0.1	9.38 ± 0.1	8.9 ± 1.1
Enzymatic antioxidant defenses			
Superoxide dismutase (percentage of inhibition)	58.9 ± 0.3	71.5 ± 0.6	72.6 ± 0.4
Catalase (nmol/min/mg of protein)	56.8 ± 0.2	72.7 ± 0.4	74.1 ± 0.5
Glutathione peroxidase (µmol/min/mg of protein)	1.63 ± 0.01	1.63 ± 0.01	1.45 ± 0.009

Protein oxidation, analyzed as carbonyl group content, showed significantly lower levels (*P* < 0.05) in the SLNs with macrometastases when compared with negative SLNs. There were also significant differences (*P* < 0.05) between the SLNs with micrometastases and macrometastases, lower values of protein oxidation appearing in the SLNs with macrometastases (Figure 3(B)).

Regarding TAC, the results showed significantly higher levels (*P* < 0.01) in the SLNs with micrometastases compared with negative SLNs. Similarly, the levels were also significantly higher (*P* < 0.01) in the SLNs with macrometastases compared with negative SLNs. There were also significant differences (*P* < 0.01) between the SLNs with micrometastases and macrometastases, higher values of TAC appearing in the SLNs with macrometastases (Figure 3(C)).

### Non-enzymatic antioxidant defense system

Figure 4 shows the levels of reduced glutathione (GSH) found in the SLNs of women with breast cancer evaluated as negative, with micrometastases, and with macrometastases. The results showed significantly lower levels (*P* < 0.05) of GSH in the SLNs with macrometastases compared with negative SLNs or SLNs with micrometastases. There were no significant differences between the levels of GSH in negative SLNs and SLNs with micrometastases (Figure 4).

### Enzymatic antioxidant defense systems

Figure 5 shows the levels of antioxidant enzymes found in the SLNs of women with breast cancer evaluated as negative, with micrometastases, and with macrometastases. SOD activity showed significantly higher levels (*P* < 0.01) in the SLNs with micrometastases compared with negative SLNs. Similarly, the levels were also significantly higher (*P* < 0.01) in the SLNs with macrometastases compared with negative SLNs. No significant differences were found between the levels of SOD in SLNs with micrometastases and macrometastases (Figure 5(A)).

Similarly, CAT activity showed significantly higher levels (*P* < 0.01) in the SLNs with micrometastases compared with negative SLNs. The levels were also significantly higher (*P* < 0.01) in the SLNs with macrometastases compared with negative SLNs. There were no significant differences between the levels of CAT among SLNs with micrometastases and macrometastases (Figure 5(B)).

Finally, GPx activity showed no significant difference between the SLNs with micrometastases when compared with negative SLNs. However, GPx levels were significantly lower (*P* < 0.01) in the SLNs with macrometastases when compared with negative SLNs or SLNs with micrometastases (Figure 5(C)). None of the parameters evaluated was useful for predicting the number of lymph nodes affected.

## Discussion

Metastasis is a complex phenomenon with multiple stages, responsible for over 90% of cancer mortality (22,23). It consists of a series of biological processes that result in the spread of tumor cells from the primary tumor to distant organs. To do this, tumor cells have to perform local invasion and intravasation, survive in blood and lymphatic circulation, reach some distant organ, perform extravasation, formation of micrometastases, and metastatic colonization, and eventually form macrometastases. All these processes are managed by different genetic and/or epigenetic tumor cell alterations (24–26). Free radicals seem to be involved in almost all of these processes, modifying one or more signaling pathways (27,28). Although it is well known that high levels of free radicals can kill tumor cells—in fact, chemotherapy is based largely on the production of free radicals—often tumor cells can use the free radicals for their own benefit (29). Thus, free radicals can help processes such as cytoskeletal reorganization, regulation of signaling pathways and transcriptional processes acting in favor of cell survival, proliferation and metastasis, including the promotion of chemoresistance (30,31). Nevertheless, the metastatic process remains one of the least known aspects of cancer. In fact, prevention and therapeutic methods are mainly directed to the removal of the primary tumor and rarely against metastasis, and the SLN represents one of the first potential structures to be colonized by tumor cells.

To our knowledge, this paper analyzes for the first time in the literature the redox status (oxidative stress parameters and enzymatic and non-enzymatic antioxidant defense systems) in the SLNs of women with breast cancer, identified by the OSNA method as negative SLNs, SLNs with macrometastases, or SLNs with micrometastasis, in order to understand the pro-oxidant/antioxidant mechanisms involved in the processes of distant metastases in breast cancer and also whether these parameters could be useful as alternate or complementary staging techniques.

Thus, we have described different levels of lipid peroxidation in SLNs with micrometastases (increased) and macrometastases (decreased), a decrease in carbonyl group content in SLNs with macrometastases only, and an increase in TAC in SLNs with micrometastases and macrometastases. A decrease in the levels of reduced glutathione (GSH) also appeared in the SLNs with macrometastases only. Finally, we have also described increased levels of SOD and CAT activity in SLNs with micrometastases and macrometastases and decreased levels of GPx activity in SLNs with macrometastases but not with micrometastases. Thus, it is confirmed that cancer in general and the metastatic processes in particular are associated with the redox status and the antioxidant defense systems, indicating that different levels of oxidative stress can promote different behaviors by tumor cells. As previously indicated, relatively low oxidative damage (i.e. a sublethal level) triggers the activation of cell signaling mechanisms that promote proliferation, migration, and invasion by tumor cells, whereas only very high levels of oxidative damage would cause cell death (32–35).

Although this study shows for the first time the redox status, analyzing oxidative stress parameters and enzymatic and non-enzymatic antioxidant defense system levels, in the SLNs of women with breast cancer, we have previously evaluated the plasma levels of these parameters (12). The results show the existence of discrepancies in both locations. Thus, in the plasma of patients with breast cancer, an increase in both lipid peroxidation and protein oxidation and a decrease in TAC were observed. These data agree with several studies that also found increased markers of oxidative stress related to lipid peroxidation (7,8,11,36,37) and protein oxidation (10,38) and a reduction in TAC (10,39) in patients with breast cancer at serum, plasma, whole blood, and red cell levels (40–43) but also in the microenvironment of the breast tumor (8,9). Therefore, our results indicate that the SLN mainly shows an antioxidant status whereas plasma reflects a pro-oxidant one. Thus, the oxidant/antioxidant mechanisms in the SLN would reflect the redox status mainly locally as a consequence of metastatic processes. Also, in the SLN, the antioxidant status promoted by metastases, as reflected by the increased TAC in micro- and macrometastases, could be due to the increase in enzymatic antioxidant defenses (SOD and CAT), which may also explain the decreases found in lipid peroxidation and protein oxidation levels in the SLNs with macrometastases. The increase in TBARS found in the SLNs with micrometastases may be due to an effect of oxidative stress in the earliest stages of metastatic colonization of the SLN, promoted by the immune response to tumor cells (33) or other metabolic events (15).

As for non-enzymatic antioxidant defense systems, a significant decrease in GSH levels has been also described in the plasma of patients with breast cancer (10,12,44). In the SLN, we also found a decrease in GSH levels with macrometastases. In general, breast cancer seems to be accompanied by a decrease of the key compound of non-enzymatic antioxidant defense systems, GSH.

Lastly, the enzymatic antioxidant defense systems are also affected in the SLN. As we mentioned above, SOD and CAT activity is increased in both micrometastases and macrometastases, which could be responsible for the increased levels in TAC, and would be the response to increased production of free radicals promoted by metastasis (33,45,46). In contrast, previous analysis in plasma (12,39,47), erythrocytes, and whole blood (41,43,48) of women with breast cancer showed decreased SOD, CAT, and GPx activity, being therefore insufficient for antioxidant protection (43,49). However, GPx activity was also reduced in the SLNs with macrometastases, which also explains the low GSH levels found at this location. Therefore, the decreased antioxidant potential that occurs in the SLN caused by reduced levels of GPx, and therefore GSH, may result from an attempt by alternate mechanisms to avoid or at least not to promote the metastatic process. In fact, it has been postulated that within the range of biomolecules damaged by reactive species, GPx may be particularly sensitive to this damage (44). In any case, low levels of GSH, both circulating and in the SLN of breast cancer patients, appear to support the hypothesis that GSH is inversely related to malignant transformation (50). Unfortunately, none of the parameters measured here could predict the number of nodes affected or proved useful for nodal staging. However, the results obtained for GPx allow us to propose it as a potential alternate biomarker for nodal staging. Due to the existence of several types of GPx which can be differentiated at a molecular level, further studies must discriminate the importance of each of them in the SLN.

Finally, we must indicate that it is not known if radiation caused by the injection of the isotope the day before the operation could influence the parameters under study. Changes have been described in several antioxidant defense systems in human and laboratory animals promoted by both ionizing and non-ionizing radiation (51), with great variability depending of the source of radiation, dose, or time of administration. However, all patients in our study received the same procedure, minimizing these putative side-effects. In any case, future research using naive lymph nodes collected from axillary clearance without isotope injection could be an interesting approach to elucidate this question.

We can conclude that in the SLN of women with breast cancer the normal redox balance is altered to an antioxidant state, shown by an increase in TAC and decreased levels of lipid peroxidation and protein oxidation, especially in SLNs with macrometastases. This antioxidant status is promoted by an increase in enzymatic antioxidant defense systems mediated by SOD and CAT, which could reflect an attempt to prevent lymph node colonization by tumor cells. The decreased levels found for GSH and GPx support the hypothesis that these antioxidant systems may be highly related to the process of metastatic invasion. Finally, although the analysis of the redox state of the SLN by simple procedures does not appear to be useful for nodal staging due to their low sensitivity and specificity, it has been demonstrated to be very useful for understanding the participation of free radicals and antioxidant systems in the metastatic process in breast cancer. We also propose molecular analysis and/or expression levels of GPx as an alternative marker suitable for lymph node staging, which needs further studies for verification.
